# Supplementary figures and images for: Morphologically and physiologically diverse fruits of two Lepidium species differ in allocation of glucosinolates into immature and mature seed and pericarp
Source: PLoS One. 2020 Aug 25;15(8):e0227528. doi: 10.1371/journal.pone.0227528 (PMC7447065; doi:10.1371/journal.pone.0227528)

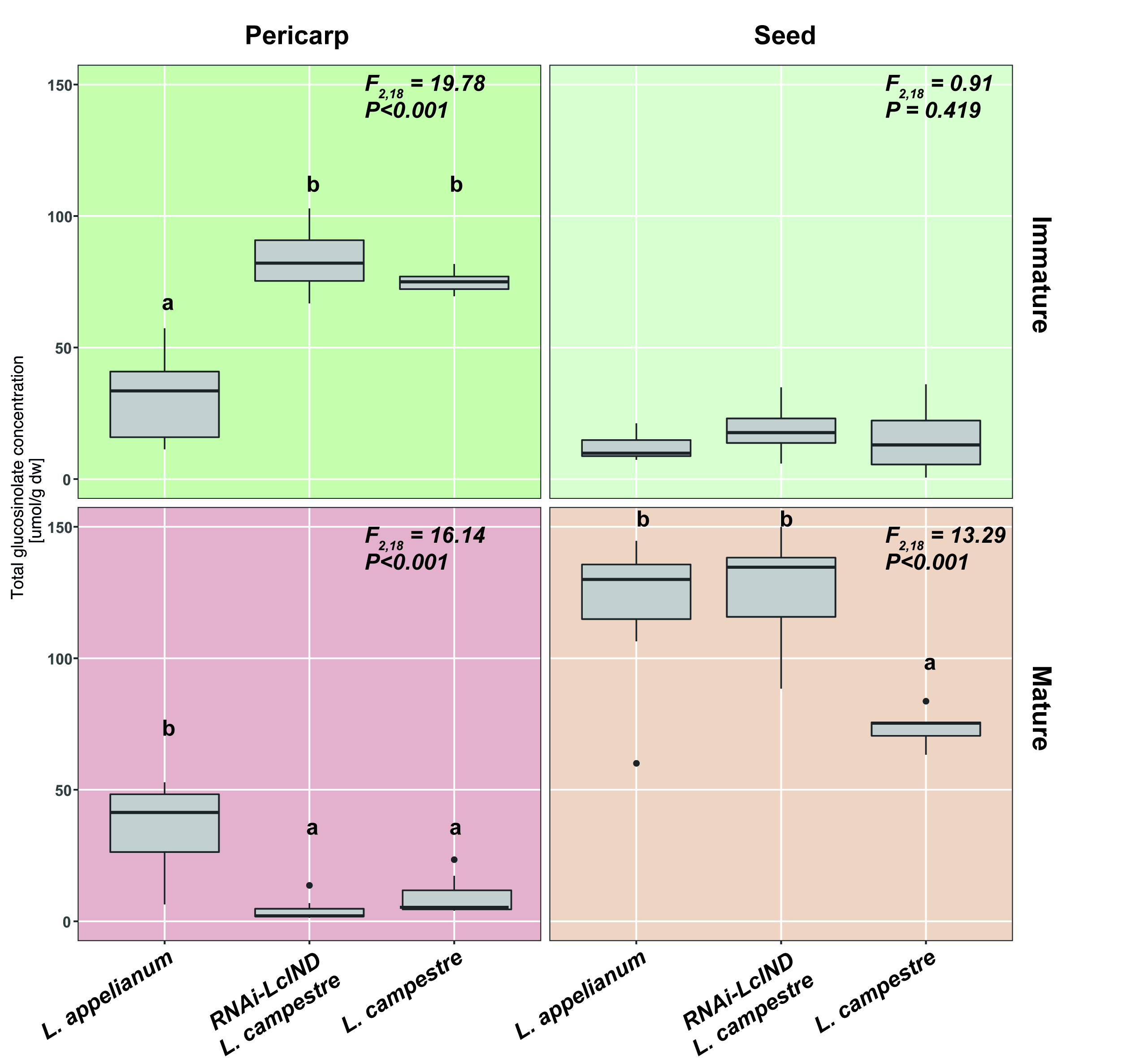

Supplement: S1 Fig — The concentrations of the total glucosinolates (μmol g-1 DW) are displayed as box-whisker plots. Boxes show the median (line) as well as the 25th and 75th percentiles, whiskers extend to the 5th to 95th percentiles and dots indicate outliers, n = 7 per species and line. Different letters within each plot indicate a significant difference in mean values in Tukey’s post-hoc analysis following ANOVA on the effects of species on total glucosinolate concentrations. (TIF) [file pone.0227528.s001.tif]
