# Supplementary material for: Morphologically and physiologically diverse fruits of two Lepidium species differ in allocation of glucosinolates into immature and mature seed and pericarp
Source: PLoS One. 2020 Aug 25;15(8):e0227528. doi: 10.1371/journal.pone.0227528 (PMC7447065; doi:10.1371/journal.pone.0227528)
Supplement: Attachment — Submitted filename: Response to reviewers_Mohammed et al.pdf [file pone.0227528.s002.pdf]

UNIVERSITÄT OSNABRÜCK

Dr. rer. nat. Samik Bhattacharya
Department of Botany
Osnabrück University
Barbarastraße 11
D-49076 Osnabrück, Germany
Tel: +49 541 969 2839
+49 152 2364 2607

To
Christophe Hano and Juergen Kroymann
Academic Editors
PLOS ONE

Date: 30. Juli 2020

Dear Dr. Hano and dear Dr. Kroymann,

Thank you very much for the review reports of our manuscript "Morphologically and physiologically diverse fruits of two Lepidium species differ in allocation of glucosinolates into immature and mature seed and pericarp" (Manuscript ID: PONE-D-19-35159R1). We hereby resubmit our revised manuscript.

Following is our detailed point-by point response to the comments from reviewers the academic editor and reviewer(s).

We also included in the submission

- A rebuttal letter that responds to each point raised by the academic editor and reviewer(s).
  - Uploaded as a separate file labeled 'Response to Reviewers_Mohammed_et_al'.
- A marked-up copy of the manuscript that highlights changes made to the original version.
  - Uploaded as a separate file labeled 'Revised Manuscript with Track Changes_Mohammed_et_al'.
- An unmarked version of your revised paper without tracked changes.
  - Uploaded as a separate file labeled 'Manuscript_ Mohammed_et_al".

We look forward to the review process.

Sincerely,

Samik Bhattacharya (on behalf of all co-authors)

Email: Samik.Bhattacharya@Biologie.Uni-Osnabrueck.de

Journal Requirements:

When submitting your revision, we need you to address these additional requirements.

1) Please amend your list of authors on the manuscript to ensure that each author is linked to an affiliation. We note that you have included affiliation numbers 1,2,3 and #a, ¶ however affiliations 1,2,3 and #a, ¶, & have authors linked to them. Please add a text to affiliation & or remove if added in error.

We amended the list of authors with linked affiliations. Previously, we tried to follow the 'instructions to authors' for assigning the symbols suggested by the journal team (e.g., first set of equal contribution by '¶', second set of equal contribution by '&', current address of an author as '#a', etc.). We recognized that the suggested system leads to more confusion than clarity. Therefore, we now used in the revised manuscript numbers for affiliations and superscripted symbols for other qualifiers ('*', corresponding author; '¶", first set of equal contributions; '¶¶', second set of equal contributions).

2) Please remove your figures/ from within your manuscript file, leaving only the individual TIFF/EPS image files. These will be automatically included in the reviewer's PDF

We removed the figures from the manuscript.

Additional comments from Editorial Board member:

a) In the abstract, the authors write 'Regarding the distribution of glucosinolate classes, high concentrations of 4-methoxyindol-3-ylmethyl glucosinolate [= 4MOI3M] were found in mature seeds of L. appelianum, while no indole glucosinolates were detected in mature diaspores of L. campestre.' (l. 37ff). But in Figure 3, the authors felt compelled to add extra panels for 4MOI3M 'due to its low abundance' (l. 294). The authors should clarify this, possibly by comparing with published values for 4MOI3M in Arabidopsis leaves or seeds (e.g., Brown et al., 2003, Phytochemistry 62, 471-481).

We rephrased the corresponding sentences in the abstract to clarify that we detected generally low concentrations of indole glucosinolates in all analyzed fruit tissues (seeds, and pericarp) compared to aliphatic and aromatic glucosinolates (lines: 38-40). The term 'relative high concentration of 4MOI3M in mature seeds' in *L. appelianum* is only relevant for comparing among the other tissue types/ species. We added a statement on the abundance of the different GLS classes as well as the values of *A. thaliana* for comparison in the discussion (lines: 432-437).

b) Fig. 3 could be improved by using a logarithmic scale for the y-axis and by putting values for L. appelianum, L. campestre and RNAi_LcIND L. campestre side-by-side for the different tissues and developmental stages.

We modified the figure 3 according to the suggestion by using logarithmic scale for the y-axis and by grouping the values of *L. appelianum,* RNAi-Lc*IND L. campestre,* and *L. campestre* side by side.

c) In the discussion, the authors write 'These results support the hypothesis that the immature pericarp in both fruit types [...] acts as a source of all GSLs and produces a comparable high level of GSLs, which are translocated to the seeds upon maturation.' - Actually, it is striking that 4MOI3M is the only indole glucosinolate that is present in pericarps or seeds of L. appelianum and RNAi_LcIND L. campestre. Based on my knowledge of the pathways (Pfalz et al., 2009, Plant Cell 21, 985-999; Pfalz et al., 2011, Plant Cell 23, 716-729; Pfalz et al., 2016, Plant Physiol 172, 2190-2203) I would also expect the presence of indol-3-ylmethyl

(I3M) and 4-hydroxy-indol-3ylmethyl (4OHI3M), the precursors of 4MOI3M, if the pericarp were producing indole glucosinolates. In fact, the absence of I3M and 4OHI3M in pericarp and seeds strongly suggests that 4MOI3M is biosynthesized elsewhere and then transported to pericarp and, subsequently, seeds.

Thank you for pointing this out. We added now a statement in the discussion: "However, for the investigated *Lepidium* species we cannot exclude the alternative explanation that GLSs are biosynthesized elsewhere and then transported to the pericarp and subsequently to the seeds" (lines: 389-391). We do not restrict this to the indole GLS, but rather keep the statement general, as this may be the case for all investigated GLSs.

Reviewers' comments:

Reviewer's Responses to Questions

Comments to the Author

1. If the authors have adequately addressed your comments raised in a previous round of review and you feel that this manuscript is now acceptable for publication, you may indicate that here to bypass the "Comments to the Author" section, enter your conflict of interest statement in the "Confidential to Editor" section, and submit your "Accept" recommendation.

Reviewer #2: All comments have been addressed

Reviewer #3: (No Response)

2. Is the manuscript technically sound, and do the data support the conclusions?

The manuscript must describe a technically sound piece of scientific research with data that supports the conclusions. Experiments must have been conducted rigorously, with appropriate controls, replication, and sample sizes. The conclusions must be drawn appropriately based on the data presented.

Reviewer #2: Yes

Reviewer #3: Partly

3. Has the statistical analysis been performed appropriately and rigorously?

Reviewer #2: Yes

Reviewer #3: Yes

4. Have the authors made all data underlying the findings in their manuscript fully available?

The PLOS Data policy requires authors to make all data underlying the findings described in their manuscript fully available without restriction, with rare exception (please refer to the Data Availability Statement in the manuscript PDF file). The data should be provided as part of the manuscript or its supporting information, or deposited to a public repository. For example, in addition to summary statistics, the data points behind means, medians and variance measures should be available. If there are restrictions on publicly sharing data—e.g. participant privacy or use of data from a third party—those must be specified.

Reviewer #2: Yes

Reviewer #3: Yes

5. Is the manuscript presented in an intelligible fashion and written in standard English? PLOS ONE does not copyedit accepted manuscripts, so the language in submitted articles

must be clear, correct, and unambiguous. Any typographical or grammatical errors should be corrected at revision, so please note any specific errors here.

Reviewer #2: Yes

Reviewer #3: No

6. Review Comments to the Author

Please use the space provided to explain your answers to the questions above. You may also include additional comments for the author, including concerns about dual publication, research ethics, or publication ethics. (Please upload your review as an attachment if it exceeds 20,000 characters)

Reviewer #2: The authors considered the majority of my comments in the manuscript, however, the response letter seems to be more convincing that the manuscript revised text regrading my questions. It means authors amended the text very conservatively and sparingly. Anyway, I think this paper is now suitable for publication, after improvement of the resolution of Fig. 3 that is still very low.

We have included more clarification in this revised version. We also reformatted the figure 3 following the suggestion of one of the reviewer and enhanced the resolution.

Reviewer #3: Mohammed et al described an interesting study toward explaining the different patterns of glucosinolates allocation into immature and mature seed and pericarp in morphologically and physiologically diverse fruits of two Lepidium species. They revealed that the concentration of glucosinolate not changed both in the immature and matured indehiscent pericarps of L. appelianum, while significant decrease in the dehiscent L.campestre and indehiscent RNAi-LcIND L. campestre. It is difficult to understand the link between pericarps dehiscent with glucosinolate concentration, and also the glucosinolate concentration and seeds dormancy. The allocation of different GSLs within seeds and pericarps of dehiscent and indehiscent fruits of Brassicaceae has been reported. Although there are no substantial flaws of the described work, its concept and methodology as well as the obtained results contain fewer exciting points.

We elaborated the introduction (lines: 58-65) and discussion (lines: 398-404, 486-498) about the relevance of defence compounds with the specific seed dispersal characters (e.g. dehiscence/ indehiscence). We established in our previous publication (Plant Cell and Environment 42, 1381-1392, 2019) that defence compounds in *Aethionema* are concentrated in the tissues/diaspores according to their defence requirements under natural conditions. Seed dispersal mechanisms play an important role in determining such defence requirements. Dehiscent fruits expose the seeds upon maturity and thus require better protection for seeds than for pericarps against potential natural enemies. In contrast, in indehiscent fruits, the pericarp needs to be provided with an enhanced defence than the enclosed seeds.

In the present manuscript, we explored if similar relationship between dehiscence/indehiscence and the GSL distribution in the diaspores exist in another Brassicaceae species, *Lepidium.* To test the hypothesis, we chose *L. appelianum* (indehiscent fruit), *L. campestre* (dehiscent fruit), and genetically transformed *L. campestre*, which produce indehiscent fruits. While the results are not as dramatic as in our previous findings in *Aethionema* on the GSL distributions, we discussed the possible reasons of our results in the ecological context of the two members of Brassicaceae (*Lepidium* and *Aethionema*).
